# An Elementary Cause of Anisocoria in Intensive Care Unit

**DOI:** 10.5005/jp-journals-10071-23213

**Published:** 2019-07

**Authors:** Shreya Das Adhikari, Raunaq Chakraborty, Sukhyan i Kerai, Mohammad Shoaib Budoo

**Affiliations:** 1-4 Department of Anesthesiology, Maulana Azad Medical College and Lok Nayak Hospital, New Delhi, India

## Abstract

**How to cite this article:** Adhikari SD, Chakraborty R, Kerai S, Budoo MS. An Elementary Cause of Anisocoria in Intensive Care Unit. Indian J Crit Care Med 2019;23(7):346.

Sir,

Sudden onset anisocoria in critical care setting can be ominous signifying various neurological conditions like uncal herniation due to intracerebral hemorrhage, hypertension or tumour, Adie's tonic pupil, glaucoma or iritis. But about 20% cases can be congenital^1^ or pharmacological. This awareness can prevent unnecessary consultation and neuro-imaging, hence reduce hospital charges. Here, we present a case of unequal papillary size following ipratropium nebulisation in a tertiary care hospital.

A 45-year-old obese female patient underwent vaginal hysterectomy performed under spinal and epidural anesthesia. She had hypertension, which was well controlled on oral drugs, history suggestive of obstructive sleep apnea (OSA) and recent upper respiratory tract infection (URI). Postoperatively, she was kept under observation in high dependency unit (HDU) in view of OSA. After a few hours of management in HDU, she developed blurry vision and had unequal pupils, which were absent in her initial examination. Immediate neurology consultation was sought. She had no signs of raised intracranial pressure or any cranial nerve pathology. An ophthalmological examination showed that best corrected visual acuity was 6/6 in both eyes, and intraocular pressure was within the normal range. The right pupil was larger than left and reaction to light exposition was slower and incomplete (Fig. 1). A fundus examination showed normal posterior segment bilaterally. As patient had recent URI she was receiving nebulization with ipratropium bromide and budenoside. It was later noticed that the nebulization mask was not fitted properly and the vapors were leaking, directed more toward right eye. After 3 hours of discontinuation of nebulization, the size of the pupil gradually decreased and was back to normal size equal to left pupil within 24 hours.

Sympathetic adrenergic stimulation causes constriction of the iris dilator and subsequent pupillary dilatation; whereas, parasympathetic stimulation mediates iris sphincter and pupillary constriction. Nebulized ipratropium bromide is one of the most commonly used medications in the ICU. It is a derivative of atropine that directly antagonizes acetylcholine at the muscarinic cholinergic receptor present on the iris sphincter. Other medications known to cause mydriasis include atropine, scopolamine, amphetamines, and serotonergic medications. Nebulised ipratropium may be cause of unilateral mydriasis from topical administration or accidental spillage of ipratropium bromide aerosol or droplets into the eye from a poor-fitting mask^2–4^ or broken nebulizer circuit.^5^ Systemic absorption would cause bilateral dilatation. The dose of the medication and the frequency of administration may also play a role in inducing mydriasis. While systemic medications cause bilateral mydriasis, direct ocular inoculation with topical medications can cause unilateral mydriasis. Similar incidents have been well described in numerous case reports and case series.^4,5^ However, unilateral mydriasis associated with ipratropium remains poorly recognized in the hospital setting. Moreover, confirmatory testing with pilocarpine administration (which demonstrates pupillary constriction in non-pharmacologic etiologies) can also confirm a diagnosis of pharmacologically induced mydriasis and potentially obviate need for imaging. However, neuro-imaging is suggested to be a necessity to exclude a compressive mass lesion in persistent fixed mydriasis beyond the most commonly reported time frame for pharmacologic causes.

**Fig. 1:**
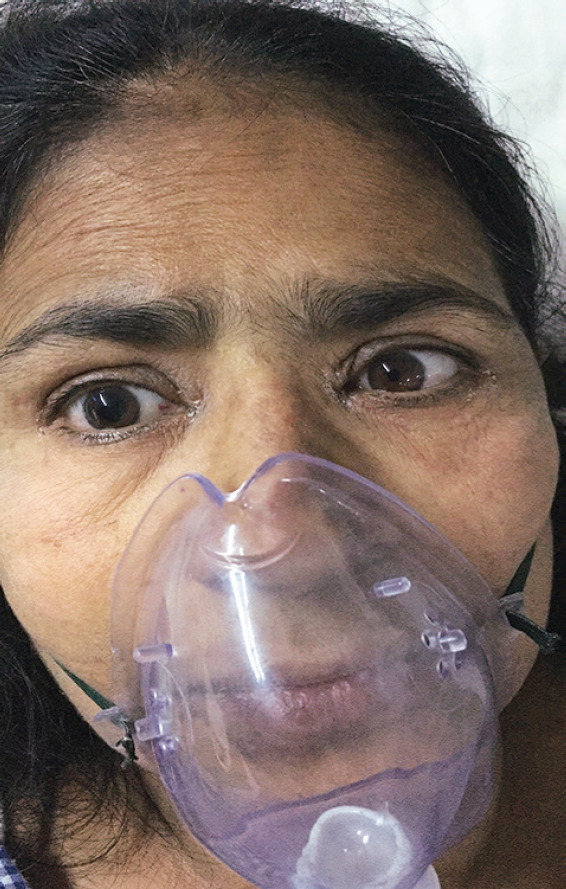
Different sized pupils with ill-fitted mask
